# Chimeras and complex cluster states in arrays of spin-torque oscillators

**DOI:** 10.1038/s41598-017-04918-9

**Published:** 2017-07-05

**Authors:** Michael Zaks, Arkady Pikovsky

**Affiliations:** 1 Institute for Physics and Astronomy, University of Potsdam, Karl-Liebknecht-Str. 24/25, 14476 Potsdam-Golm, Germany; 2 Research Institute for Supercomputing, Nizhny Novgorod State University, Gagarin Av. 23, 603950 Nizhny Novgorod, Russia

## Abstract

We consider synchronization properties of arrays of spin-torque nano-oscillators coupled via an RC load. We show that while the fully synchronized state of identical oscillators may be locally stable in some parameter range, this synchrony is not globally attracting. Instead, regimes of different levels of compositional complexity are observed. These include chimera states (a part of the array forms a cluster while other units are desynchronized), clustered chimeras (several clusters plus desynchronized oscillators), cluster state (all oscillators form several clusters), and partial synchronization (no clusters but a nonvanishing mean field). Dynamically, these states are also complex, demonstrating irregular and close to quasiperiodic modulation. Remarkably, when heterogeneity of spin-torque oscillators is taken into account, dynamical complexity even increases: close to the onset of a macroscopic mean field, the dynamics of this field is rather irregular.

## Introduction

Synchronization in large populations of self-sustained periodic oscillators occurs in many physical, biological, engineering and social systems, see recent reviews^1, 2^. The basic effect, appearance of the macroscopic mean field due to pulling together the frequencies, is described by the simple solvable Kuramoto model of sine-coupled phase oscillators^3^. However, recently it has been realized that in more involved situations also nontrivial synchronization regimes can appear, such as chaotic mean fields^4–6^, multiplicity of synchronous states^7^, glassy and Griffiths states^8, 9^, etc. Remarkably, even for identical oscillators under global (mean field) coupling, complex synchronization regimes like partial synchrony^10–12^, “chimera“s^13–15^, heteroclinic cycles^16^ have been reported. For many such regimes it is still not clear, how robust they are, and whether different dynamic states can coexist. These problems are relevant for many applications in neurosciences (see, e.g. refs 17, 18), nanospintronics (see, e.g. refs 19–21), laser physics^22^, mechanical, electrochemical, and electronic systems^23–26^, etc.

In this paper we report on nontrivial regimes of synchronization in an array of spin-torque oscillators (STOs). This consideration is relevant for applications in the generation of coherent field of significant amplitude by a coherent summation of outputs of many spin-torque nano-oscillators. Our results show that these oscillators, being more complex than the phase oscillators used in many studies, demonstrate also more complex properties of the collective dynamics.

Spin-torque oscillator is a nanoscale spintronic device generating periodic microwave (in the frequency range of several GHz) oscillations (see refs 27, 28 for an introductory review). The generation is based on the spin-transfer torque force, with which a spin-polarized electrical current (created by sending electrons through a thick layer with fixed magnetization ${\vec{M}}_{0}$) acts on a small free precessing magnet. As the electrons with spins aligned via ${\vec{M}}_{0}$ enter the free layer, a spin transfer torque acts on its magnetization, tending to reorient it, as has been theoretically predicted by Slonczewski^29^ and Berger^30^. As has been realized by Slonczewski^29^, the spin transfer torque can compensate the damping of the spin precession of the free layer, and in a constant external magnetic field a sustained oscillation (rotation of magnetization vector) takes place. After experimental observation of the generation^31, 32^, much attention has been recently attracted to synchronization of STOs; this problem is of high practical relevance, as a way to increase the output power of otherwise rather weak individual STOs^33^. For two or several STOs, mainly the couplings due to spin wave interaction^34–36^ or due to magnetic vortex interaction^37–42^ have been discussed in the literature. For a large number of STOs, the most promising way of coupling the STOs to achieve synchrony, is to connect them in serial electrically via the common microwave current^19–21, 43–46^. In ref. 19 a prototype model for such a coupling has been suggested, where *N* STOs are connected in series and are subject to a common dc current, with a parallel resistive load. The coupling is due to the giant magnetic resistance (GMR) effect, as the resistance of an STO depends on the orientation of its magnetization, so that the redistribution of the ac current between the STO array and the load depends on the the average (over the array) value of this resistance. This setup thus corresponds to the general scheme of mean-field coupling, as discussed above. However, application of the standard Kuramoto approach here^43, 47^ is rather questionable, because the STOs are highly nonlinear (see refs 44–46, 48–50) and are especially sensitive close to the homoclinic gluing bifurcation^45, 51, 52^.

In this paper we study an array of electrically coupled STOs with an RC-load. This setup is close to that considered in refs 19, 20. We will show that the STOs demonstrates a plethora of nontrivial dynamical regimes, including partial synchronization, pure and clustered “chimera” states, and clustering. Furthermore, the mean field appears to be highly irregular in a large range of parameters, making the transition to synchrony in this ensemble quite different from the usual transitions observed, e. g., in the Kuramoto model.

Before proceeding to the results, we make some remarks about the terminology used in this paper and its relation to the terms used in the literature. Most controversial is the definition of chimera states. Commonly, this term refers to situations where elements or their groups differ with respect to a certain characteristics or property. In a traditional approach, this characteristics is the individual frequency of oscillations, averaged over time: if frequencies of oscillators in a symmetric setup differ, one calls this state a chimera (see, e.g. ref. 53). Another widespread approach is based on spatial organization: one defines chimera as a profile with a coexistence of smooth and discontinuous in space patches (see, e.g. refs 54, 55). Each approach, along with merits, has its obvious limitations: the former, being restricted to the cases of continuous-time oscillators with well-defined mean frequency, is applicable neither to the ensembles of coupled maps^54, 55^, nor to the spin dynamics^56^; the latter approach assumes spatial ordering and cannot be applied to interactions in globally coupled arrays that are insensitive to distances in physical space. Below we treat a situation where none of the above approaches is applicable: we discuss globally coupled units in the regime of strong coupling where existence of a well-defined mean frequency cannot be guaranteed. Instead, below we adopt a definition of “chimera” state, based on the clustering property (the respective characteristics is the instantaneous position of the unit in its state space). This approach is applicable to globally coupled populations both of discrete maps and of continuous in time systems. We call a state in a population of identical units a “chimera” if it consists of a macroscopic cluster of units, the states of which coincide, and of a cloud of units, the states of which are different. Notice, that we put in this paper the term “chimera” into quotation marks, to emphasize the usage of a particular definition which may differ from other definitions existing in the literature.

## Results

### Basic equations

In this paper we consider an array of spin-torque oscillators, connected to the external driving current via an RC load. The external, generally time-dependent current *I*(*t*), is therefore divided between the current through the STO-array *J* and the current through the RC-load (capacitance *C*, resistance *r*) $C\dot{V}+V/r$. Here *V* = *JR* is the voltage on the array, depending on the (generally time-dependent) resistance *R* of the STO elements. This relation gives the first differential equation of the system

1 $$C\frac{dV}{dt}=-\frac{V}{r}-J+I(t\mathrm{)}.$$

The remaining equations are those for *N* STO oscillators. Each STO is described by its free-layer magnetization ${\vec{M}}_{i}$. This vector has constant unit length, and its orientation varies according to the Landau-Lifshitz-Gilbert-Slonczewski equation

2 $$\frac{d}{dt}{\vec{M}}_{i}=-\gamma {\vec{M}}_{i}\times {\vec{H}}_{eff}+\alpha {\vec{M}}_{i}\times \frac{d}{dt}{\vec{M}}_{i}+\gamma \beta J{\vec{M}}_{i}\times ({\vec{M}}_{i}\times {\vec{M}}_{0}),\quad i=1,\ldots ,N,$$

where *γ* is the gyromagnetic ratio; *α* is the Gilbert damping constant; *β* contains material parameters; *J* is the current through the STO defined above; the effective magnetic field *H*_*eff*_ contains an external magnetic field, an easy-axis field, and an easy-plane anisotropy field; ${\vec{M}}_{0}$ is magnetization of the fixed layer. Following^44^ we assume that ${\vec{H}}_{eff}={H}_{a}{\hat{e}}_{x}+({H}_{k}{M}_{x}{\hat{e}}_{x}-{H}_{dz}{M}_{z}{\hat{e}}_{z})/|\vec{M}|$. The Landau-Lifshitz-Gilbert-Slonczewski equations can be rewritten in terms of the spherical coordinates (*ϕ*_*i*_, *θ*_*i*_) determining orientation of ${\vec{M}}_{i}$, these equations are coupled via the current *J* = *V*/*R* (see Supplementary Materials for details):

3 $${\dot{\theta }}_{i}=G({\theta }_{i},{\varphi }_{i},\frac{V}{R}),\quad {\dot{\varphi }}_{i}=Q({\theta }_{i},{\varphi }_{i},\frac{V}{R}).$$

Finally, the interaction between the dynamics of the STOs and the load voltage *V* is due to the dependence of the resistance *R* on the magnetization vectors ${\vec{M}}_{i}$, according to the mechanism described in ref. 19:

4 $$R=\rho \mathrm{(1}-\varepsilon X),\quad X=\frac{1}{N}\sum _{i=1}^{N}\,\sin \,{\theta }_{i}\,\cos \,{\varphi }_{i},$$

where parameters *ρ*, *ε* depend on the resistances at parallel and antiparallel (with respect to that of the fixed layer) magnetizations. Referring for details to Supplementary Materials, we finally formulate the dynamics as a set of 2*N* equations for individual STOs, coupled via the mean magnetization field *X* through the load voltage (dimensionless variable *v*):

5 $$\begin{array}{rcl}\tau \frac{dv}{dt} & = & -v+1-\frac{v}{(1-\varepsilon X)},\quad {\dot{\theta }}_{i}=G({\theta }_{i},{\varphi }_{i},\frac{Iv}{1-\varepsilon X}),\\ {\dot{\varphi }}_{i} & = & Q({\theta }_{i},{\varphi }_{i},\frac{Iv}{1-\varepsilon X}),\\ X & = & \langle \sin \,\theta \,\cos \,\varphi \rangle =\frac{1}{N}\sum _{1}^{N}\,\sin \,{\theta }_{i}\,\cos \,{\varphi }_{i}.\end{array}$$

The main parameters that determine the coupling are the level of magnetoresistance variations *ε*, and the dimensionless time constant of the load *τ*. The main parameter determining the dynamics of the STOs is the external current *I*. Below we will study the dynamics of the array in the cases of identical and non-identical STOs. The mean magnetization *X* will be used for the visualization of the mean field behavior.

### Identical oscillators

For identical spin-torque oscillators, different cluster regimes, where the states (i.e. variables (*θ*_*I*_, *ϕ*)) of different groups of units coincide identically, are possible. The simplest of these regimes is that of full synchrony (one-cluster state), where

6 $${\theta }_{1}={\theta }_{2}=\ldots ={\theta }_{N},\quad {\varphi }_{1}={\varphi }_{2}=\ldots ={\varphi }_{N}.$$

**Figure 1:**
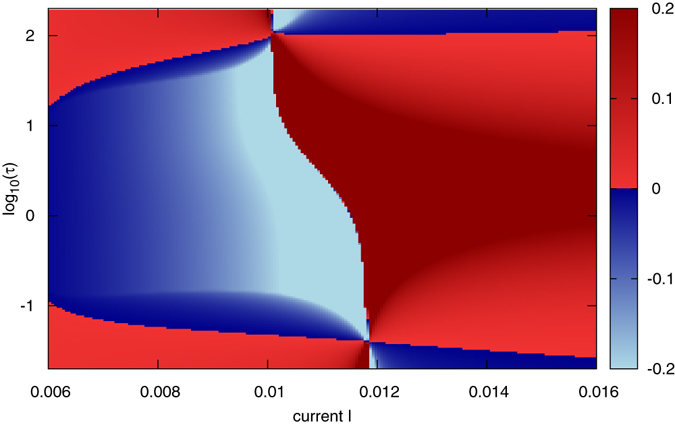
Stability of full synchrony. The evaporation Lyapunov exponent of the fully synchronous periodic state in dependence on the parameters (*I*, *τ*). This state appears in the supercritical Hopf bifurcation at *I*_*H*_ = 0.00315 (see main text), to the left from the shown range of *I*. Red domains: instability, blue domains: stability. The sharp boundary around *I* ≈ 0.01–0.012 corresponds to the homoclinic bifurcation of the periodic orbit.

The full system of 2*N* + 1 equations then reduces to a three-dimensional one; for large enough values of the external current $I > {I}_{H}=\frac{\alpha }{\beta }({H}_{a}+{H}_{k}+\frac{{H}_{dz}}{2})$ the reduced system possesses a limit cycle solution, born in the supercritical Hopf bifurcation at *I* = *I*_*H*_. Mean field in this case coincides with the field of a unit. This regime has a chance to be observed if it is stable towards “evaporation” of units from the cluster. Quantitatively, this stability is measured by the “evaporation” or “split” Lyapunov exponent^57^, see section Methods for details. A negative evaporation Lyapunov exponent means that the cluster is stable, a positive one indicates instability. We show the stability diagram in Fig. 1. One can see the large range of the parameters of the external load *τ* and of the current *I*, where the full synchrony is stable. This result is confirmed by direct numerical calculations: when the initial states are prepared sufficiently close to each other, evolution of the array ends up in the fully synchronous periodic regime.

Direct calculations however show that the full synchrony is not *globally stable*. Starting the simulations of Eqs (5) from random initial conditions, we never observed the fully synchronized state after transients. Instead, different complex configurations occur. These configurations can be classified as follows (for a practical implementation of this classification see section Methods below):

1. Partially synchronous states. In this regime the states of all oscillators are different (i.e. not a single persistent cluster is observed), however the distribution is not uniform so that the mean field *X*(*t*) performs macroscopic oscillations. These states are of the same type, as described in refs 10–12, 58 for other models of globally coupled oscillators. Noteworthy, in the partially synchronous states described in these references, the mean frequencies of all oscillators are the same. However, the notion of partial synchronization can be also applied to systems without a well-defined frequency.
2. Clustered states. In this case several clusters are built, and all the oscillators (except possibly for a few) belong to them. The effective dimension of the system is low. Clustered states of this type have been studied in the context of globally coupled maps and oscillators in refs 59–61.
3. “Chimera” states. Here one large cluster is formed, while other units remain different and build a so-called cloud. Such states have been recently reported for globally coupled oscillators in refs 13–15, 60. The dynamics here is high-dimensional, although the dimension is significantly reduced compared to the dimension 2*N* + 1 of the original system.
4. Clustered “chimera” states. Here several clusters are formed, but also a significant number of non-clustered units in a cloud is present. This regime can be considered as a combination of regimes 2 and 3.

We stress here that this classification is purely compositional one, it can be inferred from the instantaneous snapshot and does not rely on the particular dynamics of the STOs or of the mean fields; rather we discuss below how/whether dynamics is related to the compositional properties.

**Figure 2:**
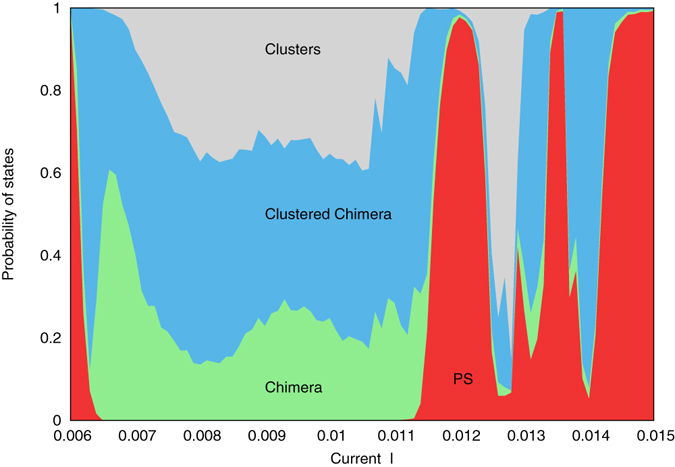
Stacked area chart representation of the frequency of occurrence of different states in an array of identical STOs. We show probability to observe different dynamical regimes: partial synchronization (PS, red), “chimera” (green), clustered “chimera” (blue), and clustered states (grey) starting from random initial conditions in an array of 200 STOs. Vertical extensions of the corresponding colors show the probabilities to observe the corresponding states in dependence on the parameter *I*. Parameters of the coupling: *ε* = 0.3, *τ* = 6.

In Fig. 2 we show the statistics of these states in a population of *N* = 200 spin-torque oscillators (this number is not very high because we needed here a large set of trials; below, when illustrating different states, we use ensembles with sizes up to *N* = 1000). Here the parameter of the external load is fixed at *τ* = 6. The computational procedure was as follows. For every checked value of *I* we localized the limit cycle corresponding to the fully synchronous state. Given a point $(\bar{\theta },\bar{\varphi },\bar{v})$ on this limit cycle, we randomly chose ca. 1800 initial conditions *θ*_*i*_, *ϕ*_*i*_, *v* from uniform distributions on the intervals $\overline{\theta } < {\theta }_{i} < \overline{\theta }+0.5$, $\bar{\varphi } < {\varphi }_{i} < \bar{\varphi }+0.5$, $\bar{v} < v < \bar{v}+0.1$, and determined the proportions of those conditions that, after a transient time of 2000 characteristic periods, ended up in one of the four states described above.

One can see in Fig. 2 that there are regions where the partial synchronous states dominate, and a large region $0.0063\lesssim I\lesssim 0.0115$ where partial synchrony is never observed, but instead clustered and “chimera” states are dominant. Remarkably, in this region the fully synchronized state is stable, so that possibly this stability is inherited by sufficiently big clusters.

**Figure 3:**
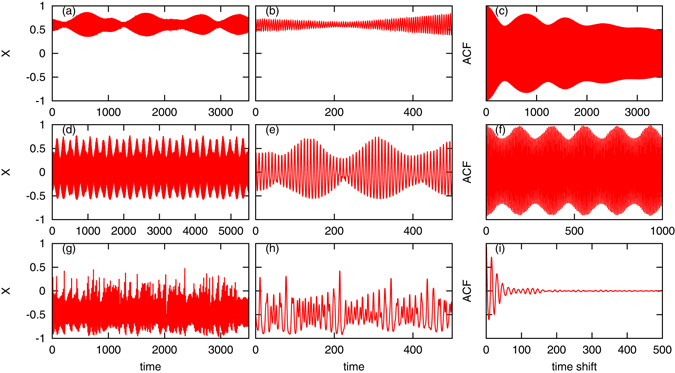
Different nontrivial dynamical regimes of the mean field *X*(*t*). Panels (a,b,c): *I* = 0.006; panels (d,e,f): *I* = 0.008; panels (g,h,i): *I* = 0.012. All data are for an array of *N* = 1000 oscillators with parameters *ε* = 0.3 and *τ* = 6. Panels (b,e,h) show details of evolution on a shorter timescale. Panels (c,f,i) show the corresponding normalized autocorrelation functions, see section Methods for the details of their calculation.

Above we characterized *compositional* properties of the complex states, by classifying them according to cluster structure; next we describe *dynamical* properties of the evolution of the mean field. For the most of the values of the current *I* presented in Fig. 2, nontrivial aperiodic dynamics of the mean field *X*(*t*) has been observed. We show three typical examples in Fig. 3. To make the time dynamics clear, we present it on two panels, at a long and at a relatively short time scales. Characterization of irregularity of the mean field in a system of many interacting units is not an easy task. Indeed, here the usual methods suitable for low-dimensional systems, such as calculation of Lyapunov exponents and of dimension are hardly applicable. Therefore we characterize regularity of the mean field by calculating the autocorrelation function (see section Methods for details). For small values of the current *I*, a very slow irregular modulation of the oscillations is observed (Fig. 3, panels (a,b)). The autocorrelation function (panel (c)) slowly decays in this case. For larger values of *I* the modulation is quite ordered, close to a quasiperiodic regime with two frequencies (Fig. 3, panels (d,e)). Here the autocorrelation function (panel (f)) returns nearly to 1, as one expects for quasiperiodic processes. For even larger values of the current, the dynamics is strongly irregular (Fig. 3, panels (g,h)). Here the autocorrelation function (panel (i)) rapidly decays nearly to zero.

**Figure 4:**
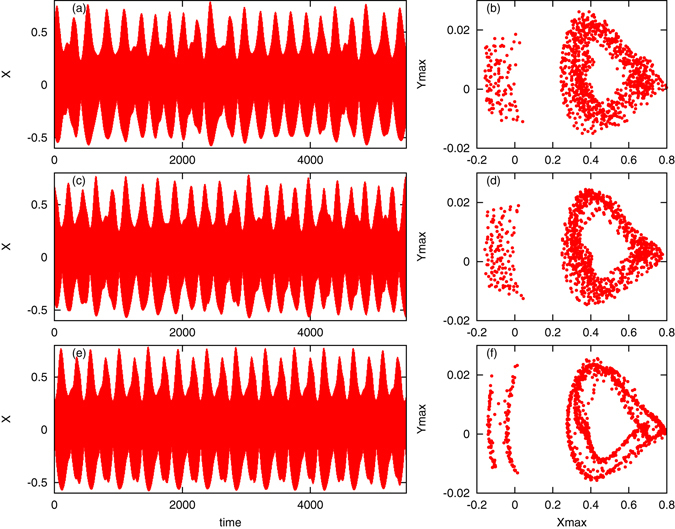
Relation between compositional and dynamical properties of the mean field. We show behaviors of the mean field *X*(*t*) for *I* = 0.008 and three compositionally different states. Panels (a,b): “chimera” state; panels (c,d): clustered “chimera” state; panels (e,f): clustered state. Panels (a,c,e) show time evolution of the mean field *X*(*t*). Panels (b,d,f) show Poincaré maps: values of the mean fields *X* and $Y=\langle \cos \,{\theta }_{i}\,\sin \,{\varphi }_{i}\rangle$ at the moments where *X* reaches local maxima. We stress here that the states are classified (“chimera”, clustered “chimera”, clustered) not according to the behavior of the mean field, but through the analysis of the instantaneous states of the units in the population, as defined above and described in section Methods.

A natural question is whether the dynamical properties of the array of STOs are related to the compositional ones. We have found that this relation is very weak. For example, regimes (a,b) and (e,f) in Fig. 3, while being both compositionally partial synchronous states, demonstrate in one case quite regular dynamics and in another case rather irregular one. The case, where for the same value of the parameters different structures are possible (like in panels (c,d) in Fig. 3) requires special consideration. Here, for *I* = 0.008, according to diagram Fig. 2, clusters, clustered “chimera”, and “chimera” states can occur. In Fig. 4 we compare the dynamical regimes for these three compositional states. Together with the time course of the mean field, we present in the right panels the Poincaré maps. Recall that a quasiperiodic attractor with two incommensurate frequencies would be seen as a smooth closed curve on the map. One can see that in all the cases the dynamics is close to a quasiperiodicity, with deviations much more noisy in “chimera” (a,b) and clustered “chimera” (c,d) regimes compared to the clustered state (e,f). This has quite natural explanation: clustered state has a rather small dimension compared to situations where there is a large cloud of unclustered oscillators, thus the effective noise is smaller.

### Nonidentical oscillators

Above we focused on the properties of an array of identical STOs. Here we study a more realistic situation, where parameters of the STOs are different. Following ref. 19, we take parameter *H*_*k*_ in the effective magnetic field ${\overrightarrow{H}}_{eff}$ to be uniformly dispersed in a range *H*_*k*0_ − Δ*H*_*k*_ < *H*_*k*_ < *H*_*k*0_ + Δ*H*_*k*_. According to general theory of the synchronization transition, e.g. from the exact solution of the Kuramoto model^1, 2^, one expects, that for a fixed coupling strength and strong enough diversity, no synchronization is observed, and this state turns into a synchronous regime with typically periodic behavior of the mean field, through a bifurcation at a certain critical value of diversity. The bifurcation is perfect in the thermodynamic limit (infinite number of oscillators) and is spoiled by finite-size fluctuations for finite ensembles.

**Figure 5:**
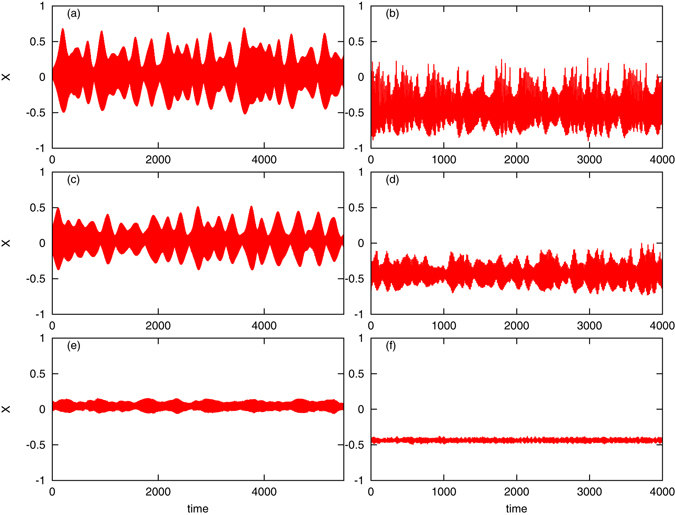
Dynamical regimes for different diversities of parameters. Panels (a,c,e): *I* = 0.008, panels (b,d,f): *I* = 0.012. Values of the parameter Δ*H*_*k*_: panels (a,b): Δ*H*_*k*_ = 0.01; panels (c,d): Δ*H*_*k*_ = 0.02; panels (e,f): Δ*H*_*k*_ = 0.04. For the regimes without diversity see panels (c,d,e,f) in Fig. 3 above.

Our numerical simulations only partially support this scenario, see Fig. 5. For large diversity in the array, indeed the oscillators do not synchronize and the mean field vanishes (see panels e,f). However, we do not observe, for a fixed level of coupling strength, a transition to a regular, periodic mean field like in the Kuramoto model: the mean field appears to be highly irregular close to the transition (panels c,d) and even for a small diversity (panels a,b). This holds for the case where identical oscillators demonstrate erratic behavior (case *I* = 0.012, panels b,d,f), as well as for the case where for identical oscillators a regime close to quasiperiodicity is observed (case *I* = 0.008, panels a,c,e). This effect is opposite to “taming spatiotemporal chaos with disorder” reported in ref. 62. Here disorder enhances collective chaos, as is especially pronounced by comparing Fig. 3(c) with Fig. 5(a,c). A more detailed exploration of the range of parameters is presented in the Supplementary Material, Figs 3 and 4. There we show the dynamics of the mean field for different levels of disorder and for different coupling strengths. One can see there that only for weak disorder and weak coupling above the synchronization threshold, the mean field is regular; otherwise irregular variations are observed. We have to stress, however, that finite-size effects may hide or destroy regularity of oscillations, therefore more detailed studies of very large arrays are needed to clarify this observation.

## Discussion

In this paper we studied complex collective regimes in an array of spin-torque oscillators coupled via a common RC load. Our main focus was on the compositional properties of the ensemble, and on the dynamical properties of the mean field. In almost all aspects, these properties are very much different from those in the standard models of globally coupled periodic oscillators, like the Kuramoto model. We did not use for classification of the states the properties of the mean frequencies, because the STOs in some regimes demonstrate mixed mode oscillations (see Fig. 2 of Supplementary Material), for which the unique frequency cannot be defined straightforwardly.

Compositional properties can be most clearly described for the identical oscillators. Here, although stability theory predicts a possibility of a fully synchronized state, practically only complex regimes between full synchrony and full asynchrony are encountered. In a large range of parameters we observed a “chimera” state, where part of the population builds a cluster and the rest remains disperse. While this regime has been recently reported for several models^13–15^, a more complex clustered “chimera” state, with several clusters and dispersed oscillators, appears to be a novel one. Together with these two types of “chimera”, a clustered state where all oscillators belong to several clusters, is also possible. Alternatively to appearance of clusters, the array can demonstrate a partially synchronous state where all oscillators are dispersed but remain correlated forming a relatively strong mean field. We reiterate that our definition of “chimera” is not based on the comparison of mean frequencies of oscillations, as has been suggested in some recent studies. Indeed, the frequency is a convenient observable for phase oscillators (or, e.g., for those obeying differential equations on a cylinder, where rotations around the cylinder can be straightforwardly counted). In our case, every individual oscillator lives on a sphere, and it cannot be guaranteed that trajectories do form in this projection a “band attractor” allowing to count oscillations and determine the mean frequency. Proper definition of a mean frequency and its analysis in different regimes may be a subject of future work.

Dynamical properties are related to the regularity of the mean field, which is well defined for nonidentical oscillators as well. One expects that for strong diversity of the oscillator’s properties, the correlation between them will be minimal and they should sum up incoherently into a constant mean field. We confirm this property for the spin-torque oscillators as well. Close to the desynchronization threshold, usually the appearing mean field is dynamically simple (periodic in time). For example, in the case where for identical oscillators one observes complex behavior related to a heteroclinic cycle^63^, by following the transition from large diversity to the small one, in ref. 64 first a transition to small periodic oscillations was detected; these oscillations undergo secondary bifurcations to more complex states only when diversity becomes sufficiently small. For the spin-torque oscillators we observe a different scenario: here close to the transition to partial coherence where the mean field is small, we observe rather irregular dynamics. For smaller disorder, the dynamics becomes more regular, close to a quasiperiodic one, for clustered states, and remains rather irregular for the partial synchronous state. This interesting observation deserves more detailed study, where especially the role of the finite size effects should be clarified.

Finally, we discuss how general are the results described above. In this paper we focused on a particular setup, where the common load for the array of STOs is of RC-type. In refs 20, 51 an array with an RCL-load has been considered. Preliminary calculations for this case show, that all four types of the composition are observed in this setup as well; however the frequency of their occurrence and the dynamical properties are different.

## Methods

### Integration of equations

Simulation of the system (5) of ODEs governing the array of spin-torque oscillators is performed by virtue of the 4th-order Runge-Kutta method. In the case of identical oscillators, there is a numerical trap due to a finite precision of computer representation of real numbers: if the states of two units are closer than the accuracy of representation of numbers in double precision (16 decimal digits), these states “merge” and remain henceforth indistinguishable. This pitfall may lead to appearance of clusters which otherwise (e.g. with quadruple precision calculations) would not exist. To avoid this effect, we add a very small diversity (of the order 10^−12^) to parameters of the oscillators, thereby preventing exact coincidence of their dynamics.

### Evaporation Lyapunov exponent

To determine the stability of the cluster solution (6), one imposes a linear perturbation *θ*_1_ → *θ*_1_ + *δθ*, *θ*_2_ − *δθ*, *θ*_3_ → *θ*_3_, …, *θ*_*N*_ → *θ*_*N*_ and *ϕ*_1_ → *ϕ*_1_ + *δϕ*, *ϕ*_2_ → *ϕ*_2_ − *δϕ*, *ϕ*_3_ → *ϕ*_3_, …, *ϕ*_*N*_ → *ϕ*_*N*_ (due to the permutation symmetry such a perturbation can be imposed to any pair of oscillators). This perturbation does not influence the mean field *X* and thus also the global variable *v*, it just corresponds to a small deviation of two oscillators from the cluster. The equations for *δθ* and *δϕ* thus read

7 $$\begin{array}{rcl}\frac{d}{dt}\delta \theta  & = & \frac{\partial }{\partial \theta }G(\theta ,\varphi ,\frac{Iv}{1-\varepsilon X})\delta \theta +\frac{\partial }{\partial \varphi }G(\theta ,\varphi ,\frac{Iv}{1-\varepsilon X})\delta \varphi ,\\ \frac{d}{dt}\delta \varphi  & = & \frac{\partial }{\partial \theta }Q(\theta ,\varphi ,\frac{Iv}{1-\varepsilon X})\delta \theta +\frac{\partial }{\partial \varphi }Q(\theta ,\varphi ,\frac{Iv}{1-\varepsilon X})\delta \varphi .\end{array}$$

Integration of these linear equations together with the full system (5) yields in the standard way the evaporation Lyapunov exponent

8 $${\lambda }_{ev}=\mathop{\mathrm{lim}}\limits_{T\to \infty }\frac{1}{2}\,\mathrm{log}\,\frac{\delta {\theta }^{2}(T)+\delta {\varphi }^{2}(T)}{\delta {\theta }^{2}\mathrm{(0)}+\delta {\varphi }^{2}\mathrm{(0)}}$$

which determines whether the cluster is transversely unstable (*λ*_*ev*_ > 0) or stable (*λ*_*ev*_ < 0).

### Distinguishing different compositional states

We used the following algorithm to distinguish different compositional states. In a population of STOs, we first identified clusters. Two units with indices *k*, *m* are assigned to the same cluster if their states are sufficiently close: *d*(*k*, *m*) < *d*_*cl*_, where *d*(*k*, *m*) = |*x*_*k*_ − *x*_*m*_| + |*y*_*k*_ − *y*_*m*_|, *d*_*cl*_ is a small number (we used in calculations *d*_*cl*_ = 10^−10^), and the local observables *x*_*k*_, *y*_*k*_ are defined in the same way as the mean fields *x*_*k*_ = sin*θ*_*k*_cos*ϕ*_*k*_, *y*_*k*_ = cos*θ*_*k*_sin*ϕ*_*k*_. If no clusters were detected in the population, (but the macroscopic mean field was present, that has always been the case) the state was classified as a partially synchronous one. The “chimera” state was the state with just one cluster which however did not include all units. We defined the clustered “chimera” state as one with more than one cluster, and a sufficient (larger that 10% of all units) number of units not belonging to the clusters. Finally, a state with many clusters and possibly a small amount (less than 10% of all units) of units outside the clusters was identified as a multiclustered state. This algorithm was used to produce Fig. 2.

### Autocorrelation function and Poincaré map

The autocorrelation function of the mean field *X*(*t*) is defined as $C(\tau )=\langle \hat{X}(t)\hat{X}(t+\tau )\rangle$ where $\hat{X}=X(t)-\langle X\rangle$. Practically, the autocorrelation function was calculated via time averaging; the length of a time series was 16000 characteristic periods. Another way to characterize the complexity of the dynamics is a Poincaré map. In our case we have chosen a two-dimensional map, by plotting all the points at which the mean field *X*(*t*) reaches a maximum, on the plane (*X*, *Y*) where the mean field *Y* is defined as

9 $$Y=\langle \cos \,\theta \,\sin \,\varphi \rangle =\frac{1}{N}\sum _{1}^{N}\,\cos \,{\theta }_{i}\,\sin \,{\varphi }_{i}$$

For the periodic motion attractor of this map reduces to a point, while for a quasiperiodic motion with two incommensurate frequencies it yields a closed curve. Poincaré maps presented in Fig. 4 are close to this structure. More complex states (quasiperiodic ones with many incommensurate frequencies and chaotic ones) result in a cloud of points lying on a fractal set (for a low-dimensional chaos) or without any structure.

## Electronic supplementary material

Supplementary material
