# Supplementary material for: Chimeras and complex cluster states in arrays of spin-torque oscillators
Source: Sci Rep. 2017 Jul 5;7:4648. doi: 10.1038/s41598-017-04918-9 (PMC5498578; doi:10.1038/s41598-017-04918-9)
Supplement: Supplementary file 1 — Supplementary material [file 41598_2017_4918_MOESM1_ESM.pdf]

Chimeras and complex cluster states in arrays of spin-torque oscillators: Supplementary material

Michael Zaks[1] **and Arkady Pikovsky**[1,2,*]

[1]Institute for Physics and Astronomy, University of Potsdam, Karl-Liebknecht-Str. 24/25, 14476 Potsdam-Golm, Germany
[2]Department of Control Theory, Nizhny Novgorod State University, Gagarin Av. 23, 606950, Nizhny Novgorod, Russia
[*]corresponding author: pikovsky@uni-potsdam.de

ABSTRACT

This text contains supplementary information to the main body of the article.

Governing equations for an array of STOs with an RC load

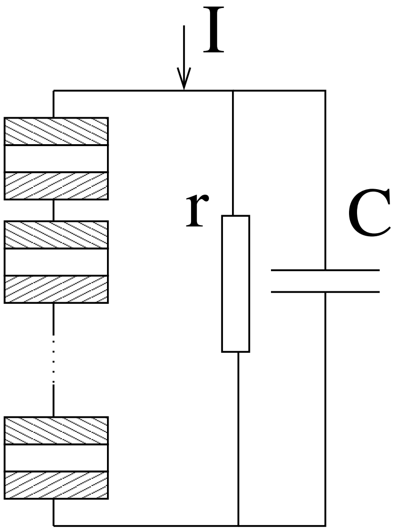

Figure 1. An array of STO oscillators with an RC load

We consider an array of STOs with a RC load as depicted in Fig. 1. The equation for the load reads

$$C\frac{dV}{dt} = -\frac{V}{r} + I - \frac{V}{R} \tag{1}$$

where R is the time-dependent resistance of the STO array, which is subject to current $J = \frac{V}{R}$. Here C and r are the capacitance and the resistance of the load. The external current I is the main governing parameter.

Each STO is described by its free-layer magnetization $\vec{M}_i$ which obeys the Landau-Lifshitz-Gilbert-Slonczewski equation

$$\frac{d}{dt}\vec{M}_i = -\gamma\vec{M}_i \times \vec{H}_{eff} + \alpha\vec{M}_i \times \frac{d}{dt}\vec{M}_i + \gamma\beta J\vec{M}_i \times (\vec{M}_i \times \vec{M}_0)\ , \tag{2}$$

where γ is the gyromagnetic ratio; α is the Gilbert damping constant; β contains material parameters; J is the current through the STO; the effective magnetic field H_{eff} contains an external magnetic field, an easy-axis field, and an easy-plane anisotropy field; $\vec{M}_0$ is magnetization of the fixed layer.

Following[1] we assume that $\vec{H}_{eff} = H_a\hat{e}_x + (H_k M_x \hat{e}_x - H_{dz} M_z \hat{e}_z)/|\vec{M}|$. Then, in spherical coordinates (ϕ, θ) the LLGS equations (2) read[1]

$$\begin{aligned} \frac{1+\alpha^2}{\gamma}\dot{\theta}_i &= U\cos\theta_i\cos\phi_i - W\sin\phi_i + \alpha S - T\ , \\ \frac{1+\alpha^2}{\gamma}\sin\theta_i\dot{\phi}_i &= -U\sin\phi_i - W\cos\phi_i\cos\theta_i - S - \alpha T\ , \end{aligned} \tag{3}$$

where

$$\begin{aligned} S = (H_{dz} + H_k\cos^2\phi_i)\sin\theta_i\cos\theta_i, \qquad T = H_k\sin\phi_i\cos\phi_i\sin\theta_i\ , \\ U = \alpha H_a - \beta J\ , \qquad W = H_a + \alpha\beta J\ . \end{aligned} \tag{4}$$

The system is closed by relating the resistance of the array R to the states of the STOs ϕ_i, θ_i. According to Ref.[2], the resistance depends on the angle δ between the magnetizations in the fixed and the free layers. In our case the magnetization of the fixed layer is along x-axis, therefore $\cos\delta = \sin\theta\cos\phi$. It is assumed that the resistance varies between value R_P (parallel magnetization, $\delta = 0$) and R_{AP} (antiparallel magnetization, $\delta = \pi$) according to

$$F(\theta,\phi) = \frac{R_P + R_{AP}}{2} - \frac{R_{AP} - R_P}{2}\cos\delta = R_0 - R_1\sin\theta\cos\phi\ ,$$

where $R_0 = \frac{R_P + R_{AP}}{2}$ and $R_1 = \frac{R_{AP} - R_P}{2}$. Then we calculate R:

$$R = \sum_{i=1}^{N}(R_0 - R_1\sin\theta_i\cos\phi_i) = \rho(1 - \varepsilon X)\ , \tag{5}$$

where

$$\rho = NR_0\ , \qquad \varepsilon = \frac{R_1}{R_0}\ , \qquad X = \langle\sin\theta\cos\phi\rangle = \frac{1}{N}\sum_{1}^{N}\sin\theta_i\cos\phi_i\ . \tag{6}$$

The final system of equations is a combination of Eqs. (1,3,4,5,6):

$$\begin{aligned} &\frac{1+\alpha^2}{\gamma}\dot{\theta}_i = U\cos\theta_i\cos\phi_i - W\sin\phi_i + \alpha S - T \\ &\frac{1+\alpha^2}{\gamma}\sin\theta_i\dot{\phi}_i = -U\sin\phi_i - W\cos\phi_i\cos\theta_i - S - \alpha T \\ &S = (H_{dz} + H_k\cos^2\phi_i)\sin\theta_i\cos\theta_i \qquad T = H_k\sin\phi_i\cos\phi_i\sin\theta_i \\ &U = \alpha H_a - \beta\frac{V}{\rho(1-\varepsilon X)} \qquad W = H_a + \alpha\beta\frac{V}{\rho(1-\varepsilon X)} \\ &X = \langle\sin\theta\cos\phi\rangle = \frac{1}{N}\sum_{1}^{N}\sin\theta_i\cos\phi_i \\ &C\frac{dV}{dt} = -\frac{V}{r} + I - \frac{V}{\rho(1-\varepsilon X)} \end{aligned}$$

We introduce new time $t' = \gamma/(1+\alpha^2)t$ and new voltage $v = V/(rI)$, then in dimensionless form we get the system (2) of the

main text.

$$\dot{\theta}_i = U\cos\theta_i\cos\phi_i - W\sin\phi_i + \alpha S - T \equiv G(\theta_i,\phi_i,\frac{Iv}{(1-\varepsilon X)})$$

$$\sin\theta_i\dot{\phi} = -U\sin\phi_i - W\cos\phi_i\cos\theta_i - S - \alpha T \equiv \sin\theta_i Q(\theta_i,\phi_i,\frac{Iv}{(1-\varepsilon X)})$$

$$S = (H_{dz} + H_k\cos^2\phi_i)\sin\theta_i\cos\theta_i \qquad T = H_k\sin\phi_i\cos\phi_i\sin\theta_i$$

$$U = \alpha H_a - \beta\frac{rIv}{\rho(1-\varepsilon X)} \qquad W = H_a + \alpha\beta\frac{rIv}{\rho(1-\varepsilon X)}$$

$$X = \langle\sin\theta\cos\phi\rangle = \frac{1}{N}\sum_1^N \sin\theta_i\cos\phi_i$$

$$\tau\frac{dv}{dt} = -v + 1 - \frac{rv}{\rho(1-\varepsilon X)}$$

where $\tau = \frac{\gamma}{1+\alpha^2}rC$ is a dimensionless time constant of the RC load.

For numerical simulations we used the following values of parameters:

$$H_a = 0.2\,, \quad \alpha = 0.01\,, \quad \beta = \frac{10}{3}\,, \quad H_{dz} = 1.6\,, \quad H_k = 0.05\,, \quad \frac{r}{\rho} = 1.$$

Mixed mode oscillations of STOs

Here we illustrate the complex form of the dynamics of one STO in a population of $N = 500$ units, in the regime of partial synchronization for $I = 0.012$ (the same parameters as for Fig. 3 (e,f) of the main text).

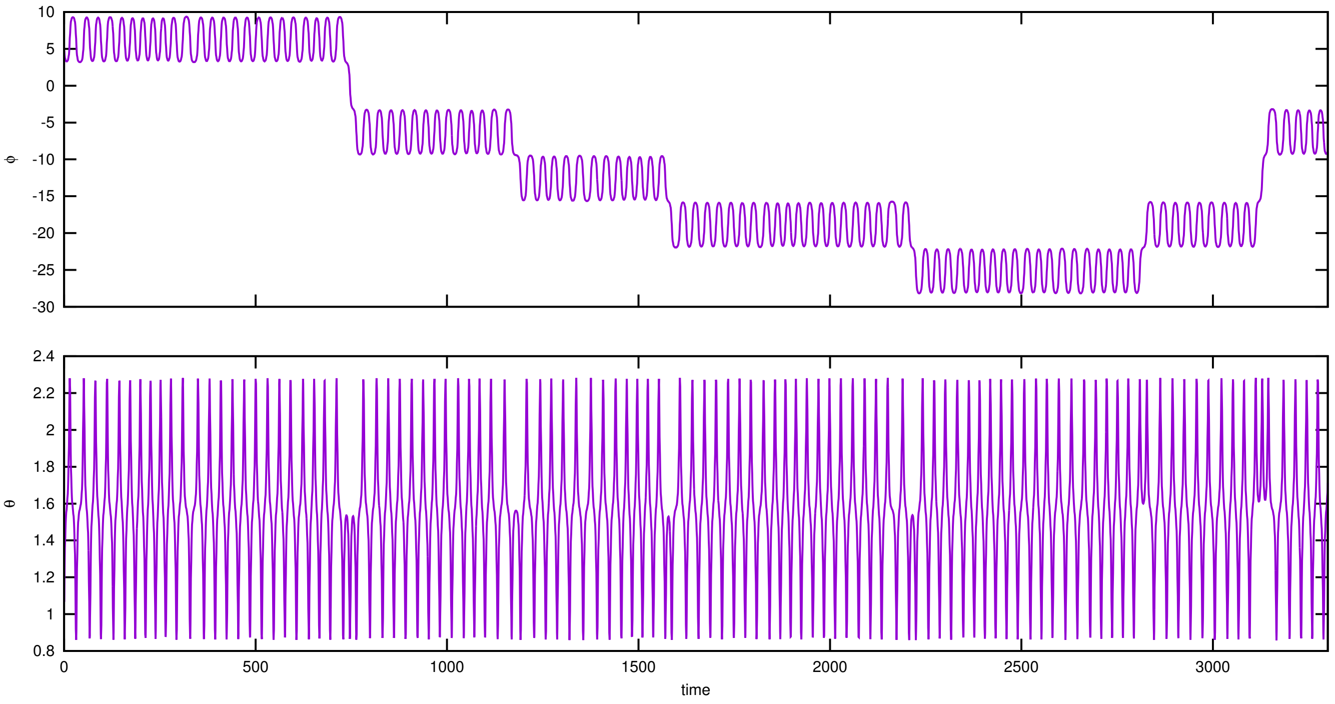

Figure 2. Time evolution of the STO variables $\theta(t)$ (bottom panel) and $\phi(t)$ (top panel). One can see that the patches where the oscillation pattern is well-defined are intermingled with switches to other oscillating mode.

1 Dynamics of the mean field for non-identical STOs, for different coupling strengths

Here we present in Figs. 3,4 how the transition to synchrony in an ensemble of nonidentical STOs occurs.

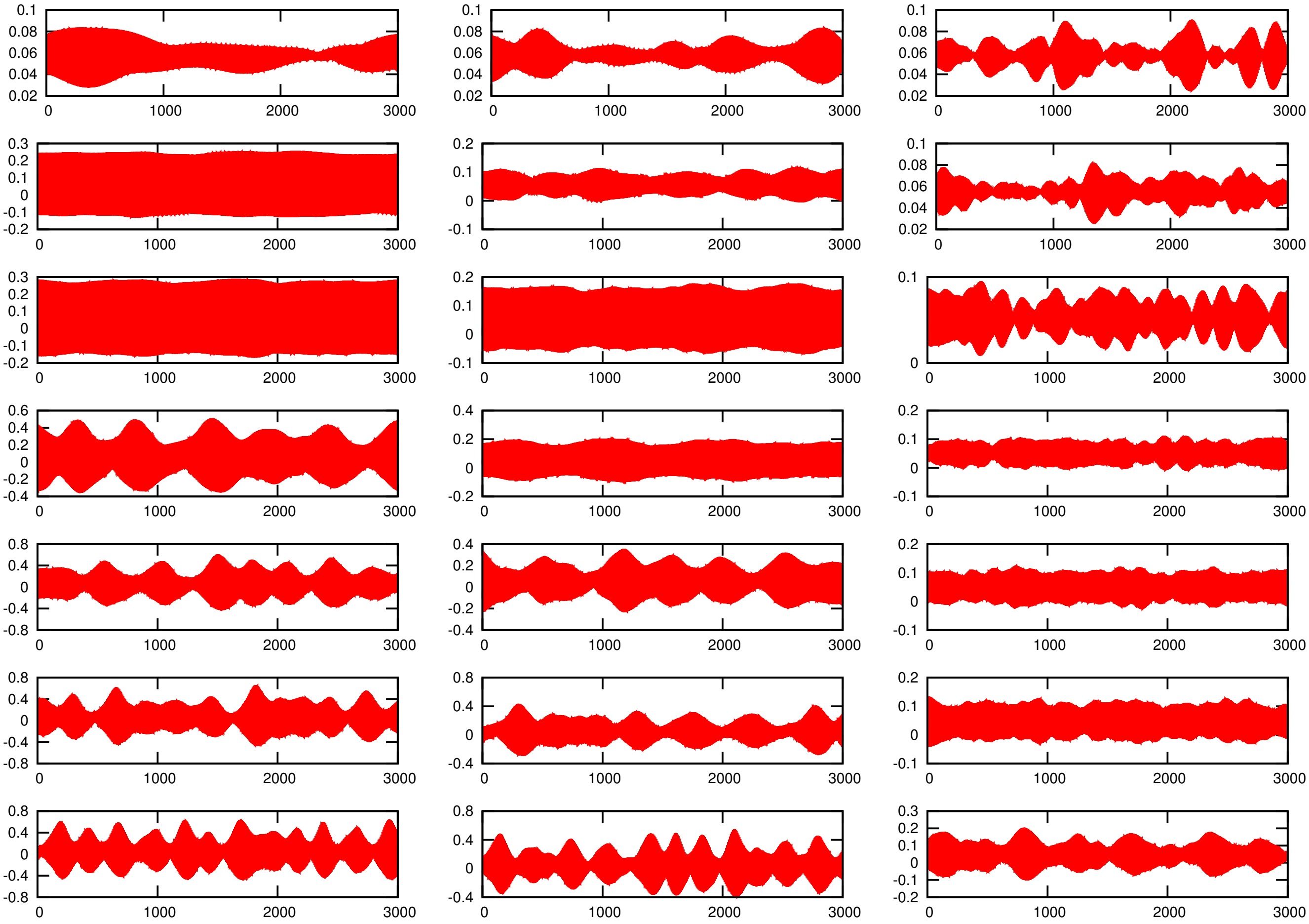

Figure 3. Dynamics of the mean field in a population of $N = 2000$ STOs with a uniform distribution of the parameter H_k, for $I = 0.008$ and different values of parameters ΔH_k (disorder) and ε (coupling strength). Left column: weak disorder $\Delta H_k = 0.01$; middle column: medium disorder $\Delta H_k = 0.02$; right column: strong disorder $\Delta H_k = 0.04$. The coupling parameter ε increases from top to bottom: $\varepsilon = 0,\ 0.05,\ 0.1,\ 0.15,\ 0.2,\ 0.25,\ 0.3$. Notice different verticales scales of the panels. The mean field is regular only for weak and medium disorder and for small coupling; here for strong coupling ($\varepsilon \geq 0.15$) the mean field is modulated. For strong disorder, significant fluctuations of the order parameter are observed for all coupling strengths.

References

1. Li, D., Zhou, Y., Zhou, C. & Hu, B. Global attractors and the difficulty of synchronizing serial spin-torque oscillators. *Phys. Rev. B* **82**, 140407 (2010). URL http://link.aps.org/doi/10.1103/PhysRevB.82.140407.

2. Grollier, J., Cros, V. & Fert, A. Synchronization of spin-transfer oscillators driven by stimulated microwave currents. *Phys. Rev. B* **73**, 060409(R) (2006).

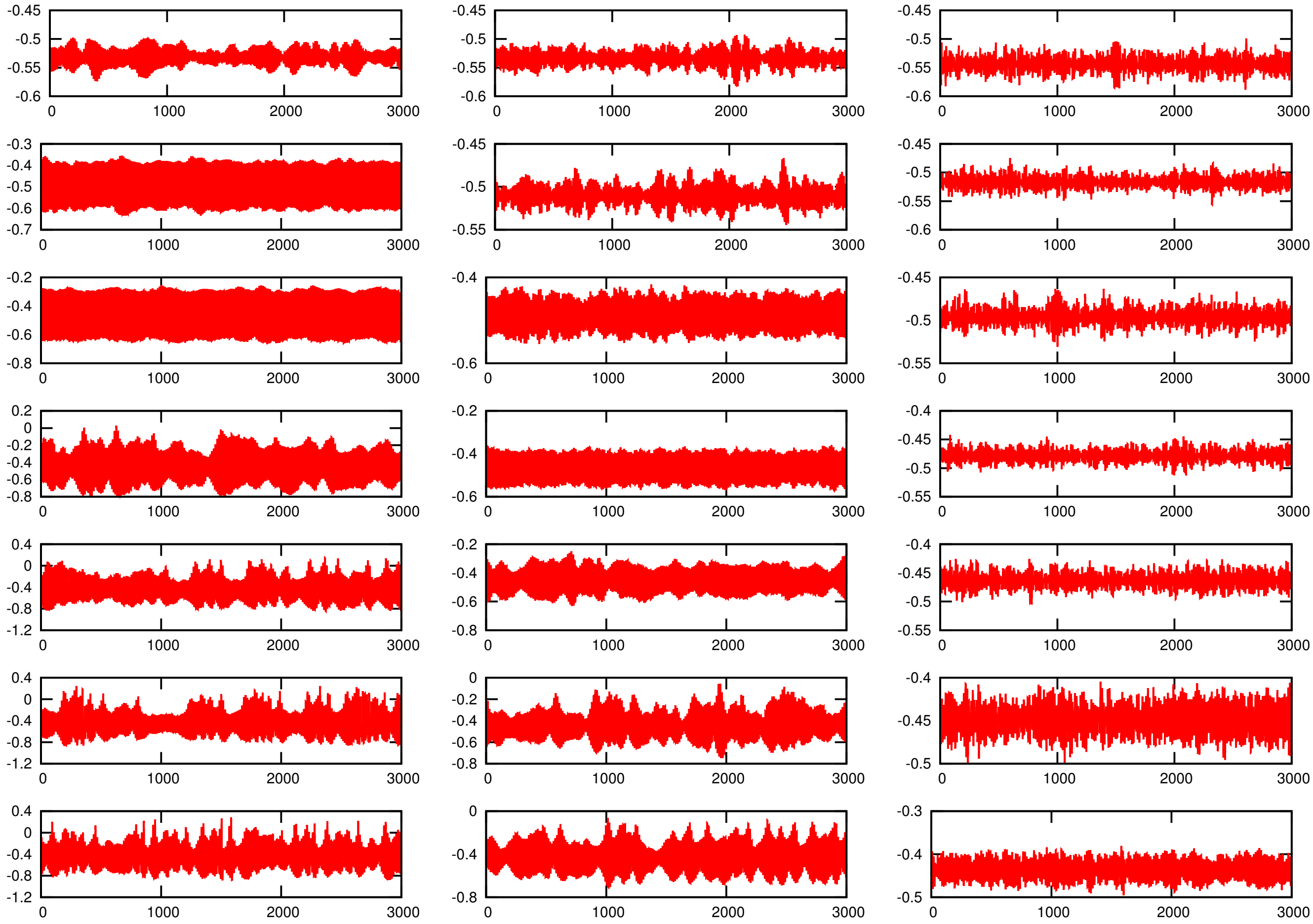

Figure 4. The same as Fig. 3, but for $I = 0.012$. Here a nearly constant mean field is observed for weak disorder and small coupling strength.
